# Prediction of the Fatigue Strength of Steel Based on Interpretable Machine Learning

**DOI:** 10.3390/ma16237354

**Published:** 2023-11-26

**Authors:** Chengcheng Liu, Xuandong Wang, Weidong Cai, Jiahui Yang, Hang Su

**Affiliations:** 1 Institute of Structural Steel, Central Iron and Steel Research Institute, Beijing 100081, China; ustbliuchengcheng@foxmail.com; 2 Material Digital R&D Center, China Iron and Steel Research Institute Group, Beijing 100081, China; tony775316@163.com (X.W.); gsgxwcwd@163.com (W.C.); yjh7646@126.com (J.Y.)

**Keywords:** fatigue strength, machine learning, symbolic regression, interpretability

## Abstract

Most failures in steel materials are due to fatigue damage, so it is of great significance to analyze the key features of fatigue strength (FS) in order to improve fatigue performance. This study collected data on the fatigue strength of steel materials and established a predictive model for FS based on machine learning (ML). Three feature-construction strategies were proposed based on the dataset, and compared on four typical ML algorithms. The combination of Strategy Ⅲ (composition, heat-treatment, and atomic features) and the GBT algorithm showed the best performance. Subsequently, input features were selected step by step using methods such as the analysis of variance (ANOVA), embedded method, recursive method, and exhaustive method. The key features affecting FS were found to be TT, mE, APID, and Mo. Based on these key features and Bayesian optimization, an ML model was established, which showed a good performance. Finally, Shapley additive explanations (SHAP) and symbolic regression (SR) are introduced to improve the interpretability of the prediction model. It had been discovered through SHAP analysis that TT and Mo had the most significant impact on FS. Specifically, it was observed that 160 < TT < 500 and Mo > 0.15 was beneficial for increasing the value of FS. SR was used to establish a significant mathematical relationship between these key features and FS.

## 1. Introduction

Metal fatigue fracture refers to the sudden brittle fracture of a metal material under the action of alternating stress after a certain number of cycles. In total, 50% to 90% of component failures are due to fatigue damage, and there is no obvious deformation of the component before fatigue failure, making it difficult to detect. This poses significant risks to actual production. Therefore, it is crucial to predict the fatigue performance of materials in advance. Traditional fatigue performance is obtained through material fatigue tests, but a single fatigue test cycle is time-consuming, resulting in a significant waste of material development costs and time. As a result, researchers have been exploring a more rapid and efficient way to predict fatigue strength (FS) [1,2]. Researchers have found that there is a certain relationship between fatigue strength and other mechanical properties of materials. For example, Wöhler et al. [3] found a linear relationship between the fatigue strength and tensile strength of materials. However, this linear relationship no longer holds as tensile strength increases further [4]. Subsequent researchers proposed other modification models [5], but they are only effective for material strength at lower levels. Additionally, material strength is influenced by factors such as composition [6], microstructure [7], and processing [8]. Establishing a predictive model between fatigue strength and other properties cannot reveal the most essential characteristics, thus having certain limitations.

With the advancement of computer science, machine learning, as an emerging technology, has been widely applied in the field of materials science. Machine learning possesses powerful fitting and predictive capabilities, allowing for the establishment of mapping relationships between material influencing factors (such as composition and processing) and target variables (such as microstructure and properties). This enables the prediction of material composition, microstructure, processing, and performance, as well as the discovery of new materials. Zhao et al. [9] developed a predictive model for the mechanical properties of cold-rolled steel using extreme random trees, achieving high-precision predictions of yield strength, tensile strength, and elongation. Lee et al. [10] utilized machine learning to design high-strength medium manganese steel, successfully developing a new type of steel with higher tensile strength than existing steel grades and almost no loss in elongation. These examples demonstrate the potential of machine learning in the materials field, enabling more accurate predictions of material properties and the discovery of novel materials with a superior performance.

This study proposes three feature-construction strategies and establishes a high-precision prediction model for FS based on machine learning. Through a series of feature-selection methods, key features influencing FS are identified. the interpretability of the prediction model is enhanced by SHAP (Shapley additional explaining) and symbolic regression (SR), and the influence mechanism of key features on FS is revealed. By adopting these strategies and utilizing machine-learning techniques, the study effectively constructs the prediction model for FS, which provides valuable insights into the influence of key features on FS.

## 2. Materials and Methods

**Figure 1:**
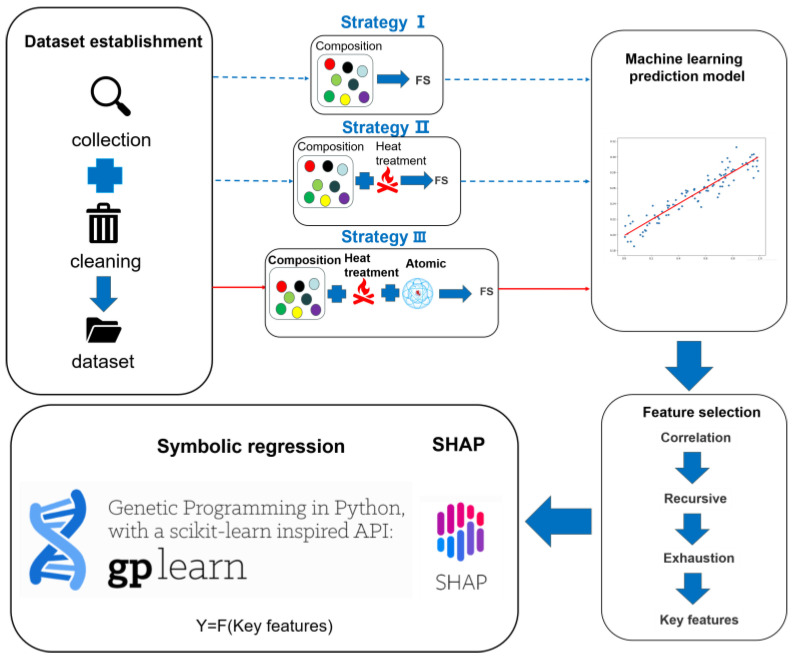
The workflow chart of fatigue strength prediction of steel based on machine learning.

Typically, the performance of materials is controlled by the combination of composition, microstructure, and processing. However, the variations and combinations of these factors result in a vast search space, making it impractical to study them individually through manual analysis. Machine learning, on the other hand, explores the hidden relationships between features and target variables, establishes quantitative models between them, and identifies the influence of features on the target variables, thereby accelerating the efficiency of materials’ research and development [11,12,13,14]. In this study, a prediction model for FS is established considering the factors of composition, processing, and microstructure. Key features are identified through feature selection, and finally, the specific expression of the prediction model is obtained using symbolic regression, clarifying the influence of each feature on FS and providing reasonable optimization suggestions. The specific workflow is illustrated in Figure 1. Initially, relevant literature and experimental data are collected and cleaned to obtain an appropriate dataset. The dataset includes information about the composition, heat treatment, and inclusion distribution of bearing steel. Three feature-construction strategies are designed based on this information. Strategy I involves inputting the composition features of steel, Strategy II involves inputting both composition and heat-treatment features, and Strategy III incorporates the atomic features of elements on top of Strategy II. The features generated by these three strategies are separately fed into different machine-learning models, and multiple metrics are used to evaluate the performance of the models in order to identify the best-performing one. Based on this, feature selection is performed on the input features. Correlation analysis, recursive feature elimination, and exhaustive methods are employed to identify the key features that have an impact on FS. Finally, SR and SHAP are used for key features to increase the interpretability of the model.

### 2.1. Data Collection and Processing

The data of this study comes from the dataset of steel fatigue strength of Japan National Institute of Materials (NIMS) [15]. The specific dataset has been attached to the Supplementary Materials in S1. The dataset consists of 26 variables, and the specific meanings and distributions of the data are shown in Table 1. The specific data are in the Supplementary Materials. The variables in the dataset include 10 composition parameters, 12 heat-treatment parameters, a rolling parameter, 3 inclusion parameters, and the target performance variable (FS). During the data-cleaning process, data with numerical anomalies were removed, and data with missing values were ignored. Ultimately, 437 sets of data were obtained for subsequent feature selection and model evaluation. To eliminate the disparity in magnitudes between different features and achieve dimensionless data, normalization was applied to the dataset. The formula for the normalization process is shown in Equation (1) [16,17].

(1) $$X^{*}=\frac{X-\min(X)}{\max(X)-\min(X)}$$

where X* represents the normalized feature, and min(*X*) and max(*X*) represent the minimum and maximum values of the original feature, respectively.

### 2.2. Machine-Learning Algorithm

Machine-learning algorithms can be categorized into classification algorithms and regression algorithms, depending on their applicability. Regression algorithms can be further classified into linear algorithms, nonlinear algorithms, and ensemble algorithms. Different algorithms are suitable for different problems, and there is no single algorithm that performs well on universal problems. Therefore, in this study, artificial neural network (ANN), elastic net regression (EN), gradient boosting machine (GBT), and bagging regression (BGR) algorithms are used to predict FS. The principle of the algorithm is introduced in the Supplementary Materials in S2. Cross-validation is employed to evaluate the performance of the models.

To evaluate the performance of different models, several commonly used statistical metrics for regression are employed, including the coefficient of determination (*R*^2^) and mean absolute percentage error (*MAPE*). *R*^2^ measures the proportion of the variance in the dependent variable that is predictable from the independent variables. It ranges from 0 to 1, where a value closer to 1 indicates a better fit between the predicted and actual values. An *R*^2^ value of 1 indicates that the model perfectly explains the variability of the target variable. The formula for calculating *R*^2^ is shown in Equation (2). MAPE measures the deviation between the predicted values and the actual values. Smaller values indicate a better predictive performance of the model. The specific formulas for calculating *MAPE* are shown in Equation (3) [18].

(2) $$R^{2}=1-\frac{\sum_{i=1}^{n}{\left(f_{i}-y_{i}\right)}^{2}}{\sum_{i=1}^{n}{\left(\overset{-}{y_{i}}-y_{i}\right)}^{2}}$$

(3) $$MAPE=\frac{1}{n}\sum_{i=1}^{n}\left|\frac{f_{i}-y_{i}}{y_{i}}\right|\times 100\%$$

where *n* represents the number of data, *y_i_* represents the actual value, *f_i_* represents the predicted value, and *ȳ_i_* represents the mean of the actual values.

In order to effectively evaluate machine-learning models, a 5-fold cross-validation approach is used to calculate the evaluation metrics during algorithm selection and subsequent feature selection. Specifically, the original data is divided into 5 subsets, with 4 subsets used for training the model and 1 subset used for validation. This process is repeated 5 times to calculate the evaluation metrics, and the average value of the 5 iterations is taken as the final performance of the model [19]. For the final model construction, a holdout method is employed where the dataset is divided into an 80% training set and a 20% test set. The model is trained using the training set and then evaluated on the test set.

### 2.3. Feature Selection

The selection of input features has a significant impact on the performance of machine-learning models. Sometimes, there may be redundant features that have an ineffective or even harmful effect on the models. Therefore, it is necessary to analyze the relationship between input features and the target variable to select the key features that influence the target variable. Common methods for feature selection in machine learning include filter methods, embedded methods, and wrapper methods [20]. In this study, filter methods and embedded methods are primarily used. Filter methods select the most predictive or informative subset of features from the original feature set, aiming to reduce dimensionality and enhance model performance. The basic idea of filter methods is to evaluate and rank features based on their statistical characteristics or correlations and select the top-ranked features as the final feature subset. In this research, the analysis of variance (ANOVA) test [21,22] is mainly adopted. ANOVA is based on the analysis of variance, which decomposes the total variance of a population into between-group variance and within-group variance. By comparing the magnitudes of these two variances, ANOVA determines whether there are significant differences in means among different groups. The between-group variance reflects the degree of difference among different groups, while the within-group variance reflects the variability within each group. If the between-group variance is significantly greater than the within-group variance, indicating that the proportion of between-group variance to total variance is relatively large, it can be concluded that there are significant mean differences among different groups. ANOVA utilizes the F-statistic for hypothesis testing. The F-statistic is the ratio of the between-group variance to the within-group variance. Based on the results of hypothesis testing, the F-value and *p*-value are obtained, where the *p*-value indicates the significance level of the difference. Generally, when the *p*-value is less than 0.05 [23], it suggests a significant linear relationship between the feature and the target label, while a *p*-value greater than 0.05 indicates no significant linear relationship between the feature and the label, and thus the feature can be discarded. Embedded methods [24,25,26], on the other hand, automatically select the optimal feature subset during the training process of the machine-learning model to improve the model performance and generalization ability. The main idea of embedded methods is to combine the feature-selection process with the model-training process. By evaluating the importance or weight of features, embedded methods embed the feature selection into the model training. In each iteration, the embedded method updates the feature subset based on the importance of features according to a predefined threshold, until a pre-defined stopping criterion or the desired number of selected features is reached.

After the selection through filter methods and embedded methods, it is often necessary to further reduce the dimensionality of the selected features. In this case, recursive and exhaustive methods [27,28,29] are commonly used for dimensionality reduction. Recursive and exhaustive methods involve calculating the performance of the model by systematically combining features, selecting the best feature combinations. However, these methods are computationally expensive due to the exhaustive search process. Therefore, they are usually employed when further feature selection is needed after the initial feature-selection steps.

## 3. Results and Discussion

### 3.1. Comparison of Machine-Learning Algorithms

**Figure 2:**
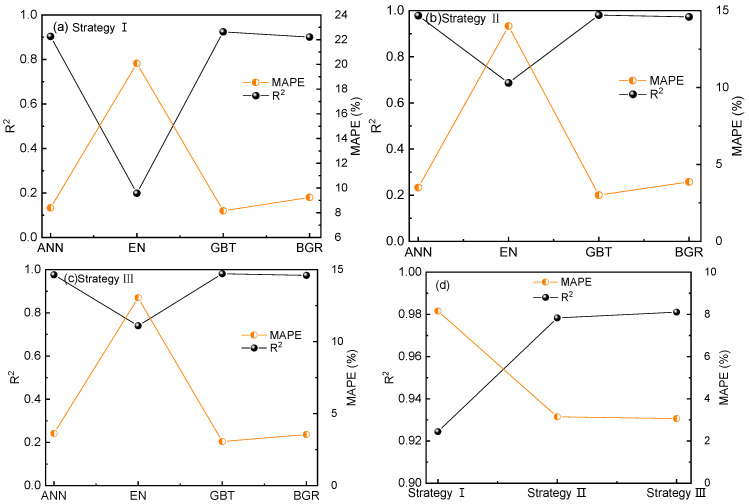
The Comparison of machine-learning algorithms under different strategies: (**a**) Strategy I, (**b**) Strategy II, (**c**) Strategy III, (**d**) different strategies in the GBT algorithm.

Figure 2 shows a comparison of the performance of machine-learning models under different feature-construction strategies. Figure 2a shows the performance comparison of different models under Strategy I. Among them, the GBT model exhibits the highest accuracy (*R*^2^ = 0.92, *MAPE* = 8.15%). However, the model accuracy is not sufficiently high, indicating that predicting FS based solely on composition is insufficient. Thus, in Strategy Ⅱ, the heat-treatment parameters are introduced. The performance of different machine-learning models under Strategy Ⅱ is shown in Figure 2b. Again, the GBT model has the highest accuracy (*R*^2^ = 0.98, *MAPE* = 3.14%). Compared to Strategy I, the model accuracy is significantly improved, indicating the important influence of heat-treatment parameters on the FS of steel. To further improve the model accuracy, Strategy Ⅲ introduces the atomic features of elements, as shown in Table 2. Figure 2c illustrates the performance of different machine-learning models corresponding to Strategy Ⅲ, where the GBT model achieves the highest prediction accuracy (*R*^2^ = 0.98, *MAPE* = 3.05%). Compared to Strategy Ⅱ, the model accuracy is further improved, indicating the effectiveness of introducing atomic features. In addition, although ANN and GBT have a similar prediction accuracy, ANN has many parameters, which leads to a long running time, so GBT is selected as the prediction algorithm. As the GBT model exhibits the highest accuracy under all three strategies, Figure 2d shows a comparison of evaluation metrics under three strategies. Different evaluation metrics all indicate that Strategy III achieves the highest accuracy.

### 3.2. Key Feature Screening

**Figure 3:**
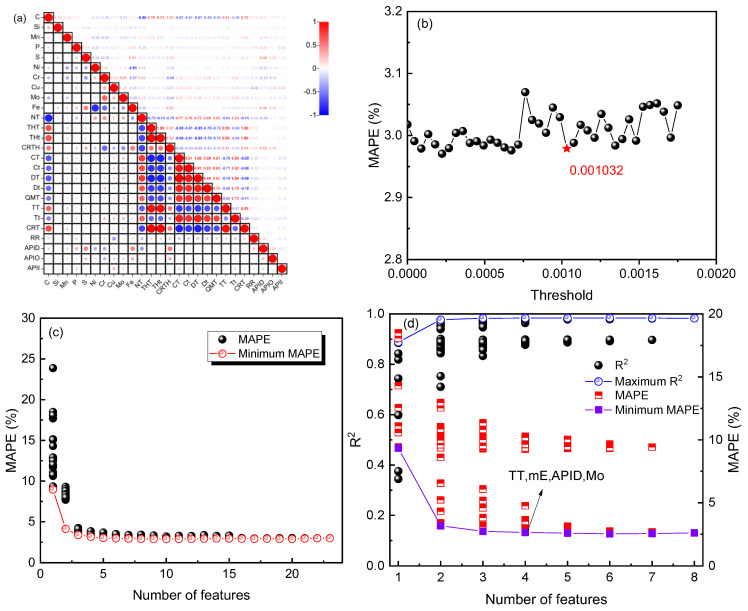
The feature-selection process: (**a**) the correlation between original features, (**b**) the influence of threshold input by embedding method on *MAPE*, (**c**) the influence of feature number on *MAPE* in recursive method, (**d**) the influence of the number of features on *R*^2^ and *MAPE* in the exhaustive method.

After determining the feature-construction strategy and machine-learning algorithm, the next step is to select the features to reduce the input dimensionality and model complexity while maintaining model accuracy. Figure 3 illustrates the feature-selection process. Figure 3a shows the correlation heatmap of the original input features, indicating a high correlation between certain features, with correlation coefficients reaching 0.95 or even 1. This suggests the need for feature correlation filtering. By conducting ANOVA tests, features with *p*-values greater than 0.05 are removed, reducing the feature dimensionality from 41 to 35. Next, the embedded method is used in conjunction with the GDB model to automatically select features, with *MAPE* chosen as the evaluation metric. Figure 3b shows the variation in *MAPE* with the input threshold of the embedded method. When the threshold is greater than 0, it indicates that features have been removed, and in this case, the model’s *MAPE* decreases, indicating the existence of redundant original input features that are detrimental to the model. When the threshold is set to 0.001032, the model’s performance improves compared to the original features (*MAPE* = 2.97%). Further increasing the threshold would lead to a decrease in model performance, so 0.001032 is chosen as the input threshold for the embedded method, reducing the feature dimensionality from 35 to 23. Figure 3c illustrates the change in the model *MAPE* with the recursive feature elimination method. As the number of input features increases, the *MAPE* decreases, indicating an improvement in model performance. When the number of features exceeds 8, the *MAPE* remains relatively unchanged, indicating that further increasing the number of features has no impact on model performance (*MAPE* = 2.90%). Figure 3d shows the impact of the number of features on the model *R*^2^ and *MAPE* through exhaustive feature selection. As the number of features increases, *R*^2^ increases and *MAPE* decreases, indicating an improvement in model accuracy. The highest accuracy is achieved when the number of features is four (*R*^2^ = 0.98, *MAPE* = 2.55%), corresponding to the feature combination TT, mE, APID, and Mo.

TT refers to the tempering temperature of steel, which mainly affects the transformation of steel microstructure during tempering and consequently influences the steel’s properties [30,31,32]. Numerous studies have already demonstrated this point. mE refers to the average electronegativity. Both reflect the ease of electron loss by atoms and indicate the strength of the metallic bonds between atoms. The mechanical strength of metals largely depends on the strength of the metallic bonds [33]. The greater the ability of an atom to provide electrons, the larger its contribution to the density of free electrons and the stronger the metallic bond. Therefore, the electronegativity can be used to estimate the strength of metallic bonds [34]. APID refers to the proportion of inclusions in steel that are deformed through plastic working. Inclusions have different compositions compared to the steel matrix. When subjected to external forces, stress concentrations occur around the inclusions. Therefore, inclusions often serve as origins of fatigue fractures and have a significant impact on the fatigue strength of steel [35,36]. The addition of Mo can effectively enhance precipitation strengthening and phase-equilibrium strengthening in steel [6], thereby influencing its properties.

### 3.3. Optimal Model Establishment

Next, an optimized machine-learning model is built using the GBT algorithm on the selected key features. During the model-building process, it is necessary to adjust the hyperparameters of the GBT algorithm to achieve optimal performance. The GBT algorithm has numerous hyperparameters, and traditional grid search or random search methods involve exhaustive searching in the parameter space, which is computationally expensive and inefficient. Bayesian optimization [37,38], on the other hand, intelligently selects parameter combinations for evaluation by modeling the relationship between parameters and the objective function, thus finding the optimal parameters faster. Bayesian optimization first requires defining the search space for hyperparameters. In this study, the parameter space is defined as follows: ‘n_estimators’ = [50, 2000], ‘learning_rate’ = [0.001, 1], ‘max_depth’ = [2, 20], ‘subsample’ = [0.5, 1], ‘min_samples_split’ = [2, 50], ‘min_samples_leaf’ = [1, 20]. The specific meaning of hyperparameters is listed in the Supplementary Materials in S2. The *MAPE* from a five-fold cross-validation is chosen as the objective function. Gaussian process regression is used to model the mapping relationship between the parameters and the objective function. Based on the current parameter combination, the objective function value is calculated. Using Bayesian theorem, the posterior distribution of parameters is computed based on the prior distribution and the objective function values. The next parameter combination to evaluate is selected based on the posterior distribution and selection strategy. This process is repeated until the specified number of iterations is reached.

**Figure 4:**
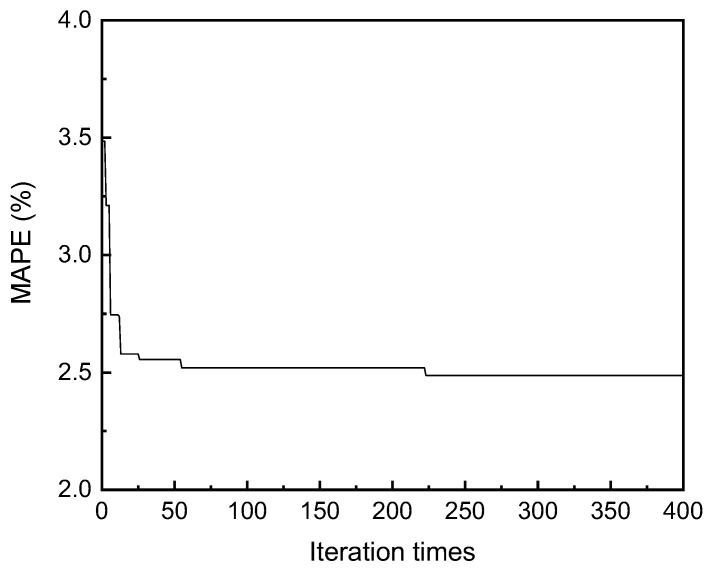
The influence of iteration times on *MAPE* in Bayesian optimization.

Figure 4 shows the change in *MAPE* with the number of iterations during the Bayesian optimization process. As the parameters are iteratively updated, *MAPE* continuously decreases, indicating the beneficial impact of Bayesian optimization on model performance. When the number of iterations reaches 230, *MAPE* no longer decreases (*MAPE* = 2.50%), and the iteration converges. The corresponding hyperparameter combination at this point is ‘n_estimators’ = 2000, ‘learning_rate’ = 0.0155, ‘max_depth’ = 13, ‘subsample’ = 0.659, ‘min_samples_split’ = 42, ‘min_samples_leaf’ = 1.

**Figure 5:**
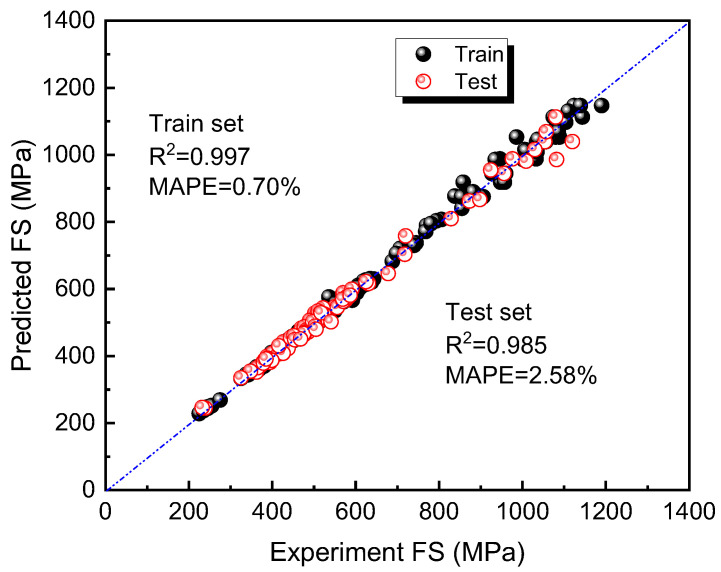
The comparison between the predicted values of the optimized machine-learning model on the training set and the test set and the experimental values of the training set and the test.

An optimized machine-learning model is built based on the hyperparameters obtained through Bayesian optimization. Figure 5 shows the comparison between the predicted and experimental values of the model on the training set and the test set. The data points on the diagonal line indicate a good predictive performance of the model (train set: *R*^2^=0.99, *MAPE* = 0.70%; test set: *R*^2^ = 0.98, *MAPE* = 2.58%).

### 3.4. Interpretable Analysis

#### 3.4.1. SHAP

To enhance the interpretability of the machine-learning model, SHAP values [39,40] are introduced. SHAP is a method that calculates the contribution of each feature by considering permutations and combinations of features to determine their impact on the prediction. SHAP employs a “feature stacking” approach, starting with cases that include only one feature and gradually adding features while computing their contributions to the results. When incorporating a new feature, it computes prediction results for all relevant feature combinations and measures the resulting changes. The SHAP value represents the average contribution of each feature to the variation in the result. To ensure equitable allocation, SHAP values consider different possibilities of feature permutations and combinations and utilize Shapley’s core to handle feature interactions. The Shapley core is a distribution scheme that guarantees the fairness principles of cooperative game theory when assigning feature contributions. By calculating the SHAP values for each feature, we can ascertain their relative importance in predicting the outcome. This interpretability enhances the comprehension of the model’s decision-making process and illuminates relationships and influences among the features. 

**Figure 6:**
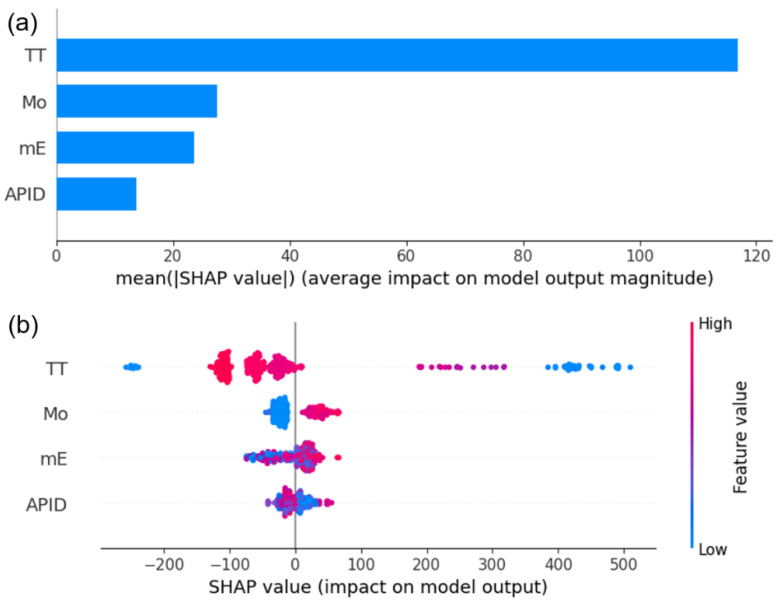
Interpretability of the SHAP value to the machine-learning model: (**a**) mean |SHAP| value (**b**) SHAP value of each sample.

**Figure 7:**
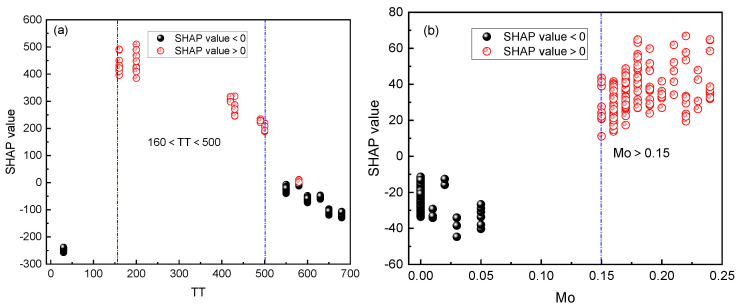
The distribution of SHAP value: (**a**) TT (**b**) Mo.

Figure 6 presents the interpretability analysis of the optimized machine-learning model using SHAP values. Figure 6a shows the distribution of the absolute values of the averaged SHAP values for each feature, where TT has the highest impact on the model’s output. Figure 6b illustrates the distribution of SHAP values for each sample, with blue representing low-data samples and red representing high-data samples. The *x*-axis represents the magnitude of the SHAP values, with positive and negative values indicating a positive or negative impact on the target variable, respectively. Taking the Mo feature as an example, when the Mo data value is high and red, the corresponding SHAP value distribution is on the positive half of the *x*-axis, indicating a positive effect on FS. Conversely, when the Mo data value is low and blue, the corresponding SHAP value distribution is on the negative half of the *x*-axis, indicating a detrimental effect on FS. Therefore, to improve the FS of the steel, it is recommended to increase the Mo content. Figure 7 displays the distribution of the SHAP values for TT and Mo. When 160 < TT < 500, the SHAP value is positive, indicating a positive impact on FS. Similarly, when Mo > 0.15, the SHAP value is positive, also contributing to the increase in FS. Conversely, when TT and Mo are in other ranges, the SHAP value is negative, impeding the increase in FS. It should be noted that the increase in Mo content is not unlimited. Excessive Mo content may have adverse effects on the properties of steel [41]. However, the dataset used in this analysis does not include such data, so it is unable to analyze the critical Mo content.

#### 3.4.2. SR

Due to the black-box nature of machine-learning models, even with the introduction of SHAP for interpretability analysis, it is still not possible to obtain specific mathematical expressions for the relationship between features and the target variable. To enhance the interpretability of the model, this study introduces SR [42,43,44], which establishes mathematical expressions between inputs and outputs. Gplearn [45,46,47], a Python open-source library based on genetic programming (GP), is utilized for SR. The process is as follows: Initially, a population of mathematical expression individuals is randomly generated, typically consisting of basic operators and functions. In this study, the basic operators include addition, subtraction, multiplication, and division, aiming to simplify the expressions as much as possible. Next, fitness evaluation is performed. The input features of the training data are plugged into the mathematical expressions to obtain predicted outputs. The fitness score of each individual is calculated by comparing the predicted outputs with the actual outputs, measuring their fit to the data. Based on the fitness scores, the selection operation is applied to choose individuals from the population for the next generation. In the selected parents, two individuals are randomly chosen for the crossover operation. The crossover operation occurs at the chromosome level of the genome, where parts of the expressions between the two individuals are exchanged to generate new individuals. Mutation may be applied to the crossover individuals to introduce new genetic variations and diversity. The mutation operation randomly modifies the mathematical expressions of individuals, such as changing constant values or replacing certain functions. The process of selection, crossover, and mutation is repeated, generating new individuals and calculating their fitness. This evolutionary iteration process gradually improves the individuals in the population to better fit the training data until the maximum number of iterations is reached, and the evolution process stops. The individual with the best fitness is selected as the final symbolic regression model. 

**Figure 8:**
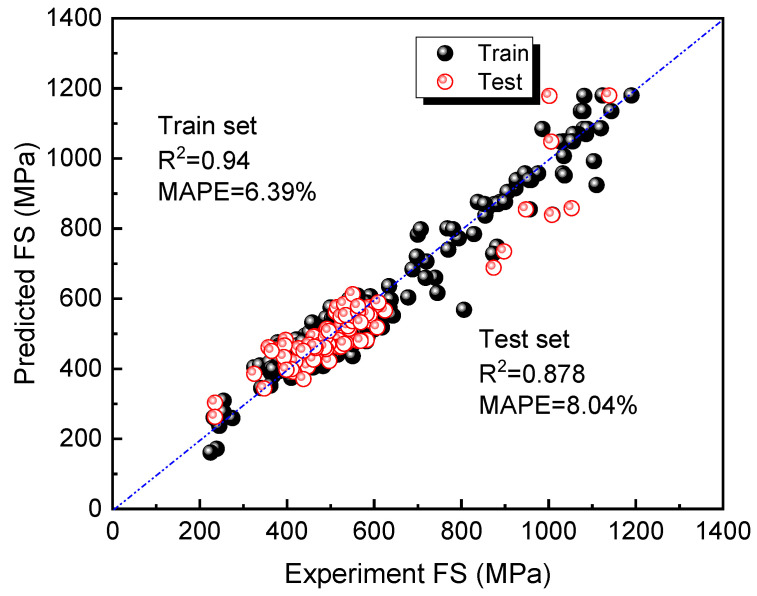
Comparison between the predicted value of SR on the training set and test set and the experimental value of the training set and test set.

The hyperparameters set for SR in this study are shown in Table 3. After 200 iterations, the expression obtained is very complicated, and the simplified formula is obtained after manual selection, as shown in Equation (4). Figure 8 compares the predicted values of FS based on Equation (4) with the experimental values in the training and testing sets. At this stage, the *R*^2^ values are both greater than 0.87 for the test and train sets, indicating the good prediction accuracy of SR. According to Equation (4), to obtain a better FS, the Mo content (Mo) and average valence electron number should be increased, while the quantity of inclusions in the steel (APID) should be decreased. Additionally, the tempering temperature (TT) should be appropriately increased.

The established SR expression can visually describe the relationship between steel features and FS, providing guidance for designing new high-FS steel grades. Compared to traditional fatigue performance research and development methods, predictive models established through machine learning and symbolic regression offer improved research and development efficiency, facilitating the exploration of higher-performance metal alloys.

(4) $$FS=(c_{0}\times Mo+c_{1}\times mE+TT\times (\frac{c_{2}\times APID}{c_{3}\times TT+Mo(c_{4}\times TT+c_{5})\times c_{6}}+\frac{c_{7}\times mE}{c_{8}\times TT}+c_{9})\times c_{10}+c_{11})$$

where *c_0_* = 45.355, *c_1_* = 47.371, *c_2_* = 3.6436, *c_3_* = 1.5942, *c_4_* = 1.9598, *c_5_* = 24.132, *c_6_* = −1.726, *c_7_* = −0.27831, *c_8_* = 0.19812, *c_9_* = 12.105, *c_10_* = −61.577, *c_11_* = 1108.7.

## 4. Conclusions

(1) Among the three feature-construction strategies and four machine-learning algorithms, the combination of Strategy Ⅲ and the GBT algorithm yields the best predictive accuracy.
(2) By using the ANOVA test, embedded method, recursive feature elimination, and exhaustive search, the key feature combination influencing FS is determined to be: TT, mE, APID, and Mo.
(3) Taking the key features as inputs, the final predictive model is established by adjusting the hyperparameters of the GBT algorithm using Bayesian optimization. At this stage, the model exhibits a good predictive accuracy (test set: *R*^2^ = 0.98, *MAPE* = 2.58%).
(4) SHAP values are used for interpretability analysis of the machine-learning model, providing insights into the contribution of each feature to the target value. When 160 < TT < 500 and Mo > 0.15, it is beneficial for increasing the value of FS. Symbolic regression is utilized to establish a mathematical expression between the key features and FS, effectively explaining the underlying mechanism of feature impact on FS.

## Figures and Tables

**Table 1:** The 26 variables of the used FS data.

	Features	Description	Max	Min	Mean
Inputs	C	wt.% of C	0.63	0.17	0.388
	Si	wt.% of Si	2.05	0.16	0.299
	Mn	wt.% of Mn	1.60	0.37	0.823
	P	wt.% of P	0.031	0.002	0.0157
	S	wt.% of S	0.03	0.003	0.0146
	Ni	wt.% of Ni	2.78	0.01	0.517
	Cr	wt.% of Cr	1.17	0.01	0.570
	Cu	wt.% of Cu	0.26	0.01	0.0677
	Mo	wt.% of Mo	0.24	0	0.0697
	Fe	wt.% of Fe	99.072	95.108	97.233
	NT	Normalizing Temperature (T)	930	825	872.299
	THT	Through Hardening Temperature (T)	865	30	737.643
	THt	Through Hardening Time (s)	30	0	25.949
	CRTH	Cooling Rate for Through Hardening (T/s)	24	0	10.654
	CT	Carburization Temperature (T)	930	30	128.855
	Ct	Carburization Time (s)	540	0	40.502
	DT	Diffusion Temperature (T)	903	30	123.699
	Dt	Diffusion time (s)	70	0	4.843
	QMT	Quenching Media Temperature (T)	140	30	35.491
	TT	Tempering Temperature (T)	680	30	536.842
	Tt	Tempering Time(s)	120	0	65.080
	CRT	Cooling Rate for Tempering (T/s)	24	0	20.814
	RR	Reduction Ratio (%)	5530	240	923.63
	APID	Area Proportion of Inclusions Deformed by Plastic Work (%)	0.13	0	0.0472
	APIO	Area Proportion of Inclusions Occurring in Discontinuous Array (%)	0.05	0	0.00339
	APII	Area Proportion of Isolated Inclusions (%)	0.058	0	0.00771
Output	FS	Fatigue Strength (MPa)	1190	225	552.90

**Table 2:** Atomic features.

Abb.	Description
mAW	mean atomic weight
mC	mean column
mR	mean row
rN	range number
mN	mean number
rAR	range atomic radius
mAR	mean atomic radius
rE	range electronegativity
mE	mean electronegativity
asve	avg s valence electrons
apve	avg *p* valence electrons
adve	avg d valence electrons
fsve	frac s valence electrons
fpve	frac *p* valence electrons
fdve	frac d valence electrons

**Table 3:** Hyperparameter in symbolic regression.

Hyperparameter	Value
population_size	3000
generations	200
tournament_size	20
stopping_criteria	0.01
p_crossover	0.7
p_subtree_mutation	0.1
p_hoist_mutation	0.05
p_point_mutation	0.1
parsimony_coefficient	0.01
const_range	(−1,1)
feature_names	[‘TT’, ‘mE’, ‘APID’, ‘Mo’]
max_samples	0.9
verbose	1
function_set	(‘add’, ’sub’,’mul’,’div’)
metric	mean absolute error
random_state	50

## Data Availability

The data presented in this study are available upon request from the corresponding author.

## Supplementary Materials

The following supporting information can be downloaded at: https://www.mdpi.com/article/10.3390/ma16237354/s1.
