# Supplementary material for: Prediction of the Fatigue Strength of Steel Based on Interpretable Machine Learning
Source: Materials (Basel). 2023 Nov 26;16(23):7354. doi: 10.3390/ma16237354 (PMC10707352; doi:10.3390/ma16237354)
Supplement: Supplementary file 1 [file materials-16-07354-s001.zip › Supplementary material S2.pdf]

Table S1 Principles of four algorithms

Algorithms	Principle
Artificial neural network (ANN)	Artificial Neural Network (ANN) is a computational model that simulates the structure and function of human nervous system, and is used to solve problems such as classification, clustering and regression. ANN is composed of multiple layers of interconnected neurons, and each layer contains some neurons. The input layer of ANN receives the original data, then transmits the data to the hidden layer, and finally reaches the output layer through a series of nonlinear transformations, resulting in the final prediction result. There are different weights in the connection between neurons, and these weights are constantly optimized in the training process to determine the best weight value, thus improving the accuracy of the model.
Elastic Net Regression (EN)	Elastic Net Regression is an extended method of linear regression, which combines the characteristics of Ridge Regression and lasso regression. Elastic network regression combines the advantages of L1 regularization and L2 regularization. L1 regularization can produce sparse solutions, that is, some coefficients are changed to zero, thus the most important features are selected. L2 regularization can reduce the amplitude of coefficients and prevent over-fitting. By adjusting the l1_ratio parameter, we can find a balance point between them.
Gradient Boosting Machine (GBT)	Gradient Boosting Machine (GBT) is an integrated learning method, which constructs a more powerful learning model by combining many weak learners. GBT gradually improves the accuracy of model prediction through iteration. In each iteration, GBT adjusts its prediction results in the last round

	according to the performance of the current model, so as to minimize the error between the current model and the real value. In the iterative process, GBT gradually improves the prediction accuracy of the model by training a series of decision trees or other basic models based on the idea of gradient descent.
Bagging regression (BGR)	Bagging regression is an integrated learning method to solve regression problems. It is based on bootstrap sampling and several basic models. In Bagging regression, firstly, the self-help sampling method is used to randomly select samples from the training set and generate multiple new training sets. Then, each new training set will be used to build a basic model independently. The commonly used basic models include decision trees, random forests and so on. Next, the final prediction result is obtained by averaging or weighted averaging the prediction results of several basic models.

Table S2 Meaning of Hyperparameter in Machine Learning Algorithm

Algorithm	Hyperparameter	Meaning
GBT	n_estimators	Number of weak learners (base learners) used
	learning_rate	The weight (or contribution) reduction coefficient of each weak learner.
	max_depth	Maximum depth of each decision tree
	min_samples_split	Minimum number of samples required to split internal nodes.
	min_samples_leaf	Minimum number of samples required for each leaf node
